# Brucellosis manifesting as reversible ataxia caused by SIADH-related hyponatremia

**DOI:** 10.1016/j.idcr.2020.e01043

**Published:** 2021-01-02

**Authors:** Mhd Baraa Habib, Omnia Hamid, Amina Bougaila, Hiba Habib, Mouhand F.H. Mohamed

**Affiliations:** a Department of Internal Medicine, Hamad Medical Corporation, Doha, Qatar; b Damascus University, Faculty of Medicine, Syria

**Keywords:** SIADH, Syndrome of Inappropriate Secretion of Antidiuretic Hormone, Sodium, Syndrome of inappropriate ADH secretion, Brucellosis, Ataxia, Cerebellar

## Abstract

Brucellosis is an infectious disease commonly presents with fever, fatigue, arthralgia, and muscle pain. Nonetheless, hyponatremia and its related symptoms have been reported as presenting features of brucellosis. Herein, we report a 56-year-old male presenting with ataxia and SIADH-related hyponatremia induced by brucellosis. The ataxia improved following hyponatremia correction. Up to our knowledge, this is the first case of reversible cerebellar ataxia occurring in the setting of brucellosis-related SIADH.

## Introduction

Brucellosis is a common bacterial zoonotic disease caused by Brucella, a gram-negative, aerobic coccobacilli. It affects mammalian species, including humans. Human infection is predominantly caused by B. melitensis and B. abortus. It is usually acquired from contaminated dairy products or direct contact with infected animals' fluids among people in contact with animals [1,2]. Brucellosis can manifest with a variety of symptoms. Fever, sweating, and osteoarticular symptoms, hepatomegaly, splenomegaly, and lymphadenopathy are well-known manifestations. Neurological findings such as meningoencephalitis, chorea, and peripheral neuropathies were reported [1].

Syndrome of inappropriate secretion of antidiuretic hormone (SIADH) is a common cause of hyponatremia. This syndrome is characterized by euvolemic hyponatremia, low plasma osmolality, relatively high urinary osmolality, and natriuresis. A wide spectrum of diseases and disorders are associates with SIADH, including pulmonary diseases, central nervous system diseases, cancers, and medications [3]. SIADH is temporarily treated by fluid restriction. Nevertheless, treating the underlying disease is the definitive treatment [4].

Here we report a rare case of hyponatremia-induced ataxia in the context of brucellosis. The patient's neurological symptoms reversed following hyponatremia correction.

## Case report

A 56-year-old male patient works in close contact with animals and is known to have diabetes mellitus and hypertension presented with two days history of fever, fatigue, and sore throat. He received an empiric course of oral amoxicillin. A few days later, he presented to the emergency department with dizziness and progressive imbalance for two days, and persistent fever. He also had nausea and vomiting; however, without abdominal pain or recent bowel habits change.

On admission, he was febrile 38℃. Blood pressure was 140/84 mmHg, heart rate 99 beats per minute, with normal respiratory rate and oxygen saturation. Neurological examination was only remarkable for ataxic gait and dysmetria in both upper limbs. There were no clinical signs of dehydration or overload, and other systemic examinations were unrevealing. Laboratory tests showed severe hyponatremia Na 115 mEq/L (normal baseline sodium). He had normal urea and creatinine. Random blood glucose was 11.2 mmol/L. Because the patient has been having nausea and vomiting, he initially received 500 mL of normal saline. His serum sodium improved to 123 mEq/L after 6 h. Further workup revealed low serum osmolality, relatively higher urine osmolality, and urinary sodium, suggesting SIADH (Table 1); hence, he was started on fluid restriction. Thyroid-stimulating hormone and cortisol levels were normal.

**Table 1:** The patient's laboratory test results. Please proceed.

Detail	Value w/Units	Normal Range
U24 Sodium	52 mmol/24 h	40−220
Urine Osmolality	531 mmol/kg	150−1,150
Serum Osmolarity	269 mmol/kg	275−295
Glucose	11.6 mmol/L	3.3−5.5
Procalcitonin	2.34 ng/mL	
Cortisol	774.0 nmol/L	
TSH	1.09 mIU/L	0.30−4.20
FT4	14.6 pmol/L	11.6−21.9
WBC	4.0 × 10^3/uL	4.0−10.0
Hgb	14.2 gm/dL	13.0−17.0
Urea	6.9 mmol/L	2.8−8.1
Creatinine	80 umol/L	62−106
***Sodium***	***115 mmol/L***	***136−145***
Potassium	4.3 mmol/L	3.5−5.1
ALT	60 U/L	0−41
AST	65 U/L	0−40
CRP	113.4 mg/L	0.0−5.0
Brucella meletensis Titer	Positive 1:1280	
Brucella Ab IgG	Positive	
Brucella Ab IgM	Positive	

Given animal contact history, brucella serology was sent and returned positive for both IgM and IgG. Moreover, the blood gram stain revealed the growth of gram-negative coccobacilli. The chest X-ray and ultrasound abdomen showed no abnormality. Head magnetic resonance imaging was normal.

**Fig. 1:**
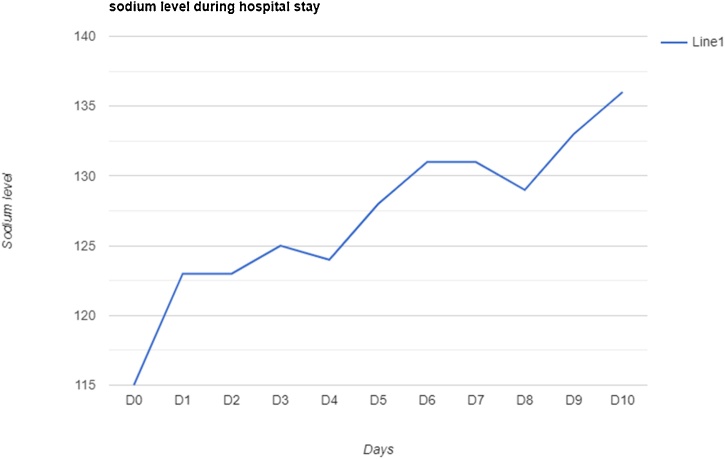
Sodium level changes during the hospital stay.

The patient was admitted as a case of brucellosis and started on oral doxycycline and intravenous gentamycin. This was associated with remarkable improvement in his ataxia, nausea, and dizziness. The mentioned symptoms subsided one day after admission except for the fever, which persisted for three days. The sodium level improved gradually and eventually became normal (Fig. 1).

## Discussion

In our case, the diagnosis of SIADH was supported by hyponatremia, low plasma osmolality, relatively high urinary osmolality, and natriuresis; additionally, ruling out other possible etiologies such as hypothyroidism and adrenal deficiency [4]. The hyponatremia responded to fluids restriction, which further supports the diagnosis of SIADH. SIADH is associated with infections, malignancy, pulmonary or neurological conditions, and medications [4]. Prevalence of SIADH in the setting of brucellosis was reported to be as high a 54 % [5]. A study done on pediatric patients with brucellosis revealed that brucellosis patients with SIADH have significantly higher levels of glucose, inflammatory markers, and liver enzymes [6]. Generally, fluid restriction and treating brucellosis are enough to manage brucellosis-related SIADH. In contrast, acute symptomatic hyponatremia warrants prompt diagnosis and treatment to avoid neurological complication [7,6].

Isolated reversible cerebellar ataxia as a presentation of hyponatremia with or without SIADH has been rarely reported [[8], [9], [10]]. This adds to the uniqueness of our case, as this is the first reported case of reversible cerebellar ataxia in the context of SIADH and brucellosis. The patient ataxia resolved completely after hyponatremia correction. The mechanism of reversible ataxia in the setting of SIADH is not well elucidated, especially in the absence of cerebral pontine myelinolysis [11,12]as in our patient [13]. Notwhistanding this, early demyelination cannot be ruled out; thus, timely diagnosis and treatment of hyponatremia and its cause are imperative to avoid short or long-term adverse sequelae.

## Conclusion

SIADH in the setting of brucellosis is not uncommon, and clinicians must be aware of this relationship. The treatment entails treating brucellosis and fluid restriction. Reversible cerebellar ataxia caused by hyponatremia is rather rare, and our case is the first to depict in the context of SIADH and brucellosis. Timely identification and management are essential to avoid adverse outcomes.

## Funding

This research did not receive any specific grant from funding agencies in the public, commercial, or not-for-profit sectors.

## Consent

Due to the COVID-19 situation and its impact on direct patient contact, only verbal consent was obtained to publish this case.

## CRediT authorship contribution statement

**Mhd Baraa Habib:** Conceptualization, Writing - original draft. **Omnia Hamid:** Writing - review & editing. **Amina Bougaila:** Writing - review & editing. **Hiba Habib:** Writing - review & editing. **Mouhand F.H. Mohamed:** Supervision, Writing - review & editing.

## Declaration of Competing Interest

The authors have no conflict of interest relevant to this case.
